# Post-Analytical Tools for the Triage of Newborn Screening Results in Follow-up Can Reduce Confirmatory Testing and Guide Performance Improvement

**DOI:** 10.3390/ijns6010020

**Published:** 2020-03-14

**Authors:** Patricia L. Hall, Angela Wittenauer, Arthur Hagar

**Affiliations:** 1 Department of Human Genetics, Emory University, Atlanta, GA 30322, USA; 2 Georgia Department of Public Health, Atlanta, GA 30303, USA

**Keywords:** newborn screening, newborn screening follow-up, post-analytical tools, performance improvement

## Abstract

Georgia uses post-analytical tools through Collaborative Laboratory Integrated Reports (CLIR) to triage abnormal newborn screening (NBS) results for follow-up. Condition specific tools are used to assign each case a risk level, which is used to guide follow-up recommendations. Follow-up recommendations include assessment by the child’s primary care provider as well as testing, either a repeat NBS or confirmatory testing. Triaging abnormal cases using these tools has been advantageous in managing the workflow for the follow-up team, as well as prioritizing cases that appropriately require more attention and resources. The initial goal in utilizing these tools was to reduce the amount of confirmatory testing, particularly for disorders where there are many false positives. We assessed the performance of these tools retrospectively for three of the most commonly detected conditions by tandem mass spectrometry in Georgia: phenylketonuria, medium chain acyl-CoA dehydrogenase deficiency and very long chain dehydrogenase deficiency. The post-analytical tools appropriately assigned all true positive cases to the higher levels of follow-up testing and reduced the level of intervention for a significant number of cases as well. Based on the experience gained from our utilization of the tools in the follow-up program, we are well situated to move forward with using the tools in a more prospective manner, and reduce the number of cases that will be reported, rather than just assigning resources appropriately at follow-up. Post-analytical tools are an improvement over trying to capture the variation in the newborn population using multiple cutoffs. It also easily identifies significant abnormalities that are unrelated to inherited disease, such as large amino acid elevations due to total parenteral nutrition.

## 1. Introduction

As a public health program, newborn screening (NBS) encompasses much more than the laboratory and bedside tests that provides the result that either reassures or refers for further analysis [1]. Recently, NBS systems have encountered increased scrutiny, and an increased emphasis has been placed on performance improvement. In this environment, it is incumbent upon NBS systems to evaluate their performance and identify opportunities for improvement [2]. The laboratory is usually the primary target for performance improvement initiatives, as the metrics are readily available and changes at the laboratory can impact the largest number of patients quickly. Post-analytical tools have been used in NBS to improve positive predictive values (PPV) in the laboratory for many disorders [3,4,5]. The current iteration of post-analytical tools through Collaborative Laboratory Integrated Reports (CLIR; www.clir.mayo.edu) have an extensive database of cases, and reference data which can be used to identify abnormal screens by comparison to confirmed cases, rather than deviation from the reference range [3]. Implementation of post-analytical tools in the laboratory setting can be dependent upon laboratory information systems and vendor support, making it a challenge for some programs which may be resource strapped. These challenges do not mean that such programs cannot still benefit from the utilization of such tools in other ways.

When there are abnormal results for a condition detected by NBS, the infant needs to be identified and recalled for follow-up. Standardized recommendations exist for testing and assessments that need to be done for specific disorders [6]. For conditions which can require immediate medical attention and intervention where there may exist a high probability of false positive (FP) results, additional triage is required to ensure infants who require follow-up receive the proper attention, CLIR tools have been important in follow-up for identifying these cases out of the cohort of FP screens. For many laboratory tests, different cutoffs, including higher “critical” or “panic” values can identify high priority laboratory results, but this approach is not always ideal for NBS, particularly for amino acid disorders. Many of the most significantly elevated values encountered in NBS are obvious FP results due to collection of a screen while an infant is receiving total parenteral nutrition (TPN) or from a central line [7,8]. All abnormal NBS results exist within the infant’s clinical context, and developing robust algorithms to identify all of these situations is difficult. For some disorders, even mild elevations are practically diagnostic (succinylacetone in tyrosinemia type I [9], or alloisoleucine in maple syrup urine disease [9,10]).

In Georgia, laboratory testing for NBS disorders is performed at the state public health laboratory. Follow-up for all disorders except hemoglobinopathies is coordinated through Emory University. Hemoglobinopathy follow-up is coordinated through the Department of Public Health. Results are transmitted from the state laboratory to the follow-up team, into a custom-built database. This database generates lists of cases which require follow-up. Follow-up protocols are developed in consultation with specialists and approved by the medical director. The follow-up team is made up of registered nurses and a genetic counselor, who provide information to primary care providers and recommend testing based on the screening results utilizing established protocols. To triage follow-up, Georgia’s follow-up team has been using post-analytical tools for over ten years. Initially, early versions of the Region 4 Stork tools were utilized to better prioritize screens that were positive for very long chain acyl-CoA dehydrogenase (VLCAD) deficiency. This decision was an attempt to prioritize cases that needed to be referred to metabolic specialists and also to reduce confirmatory testing in favor of repeat screens, and deal with the large number of carriers who have a biochemical phenotype [11]. Georgia has the same shortages of genetics professionals as the rest of the United States, and minimizing unneeded appointments is crucial. As post-analytical tools for more conditions became available, follow-up protocols were adjusted to accommodate them. In April 2018, we switched to the updated version, CLIR. This allowed for the adjustment based on age at collection and birth weight, as well as the easy creation of custom tools for our site, to account for different analytes and local protocols.

**Figure 1:**
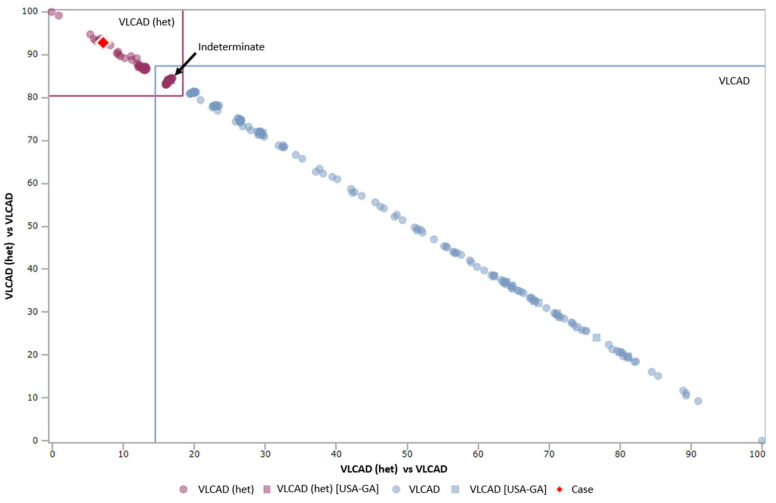
Dual scatter plot for very long chain acyl-CoA dehydrogenase (VLCAD) vs. VLCAD (heterozygous) designed to reduce false positive (FP) NBS results due to biochemical profiles in carriers. The upper left corner is confirmed VLCAD carriers cases. The bottom right corner is confirmed true positive (TP) cases of VLCAD deficiency. The square in the middle is an indeterminate zone where carriers cannot be distinguished from TP cases. In this example, the red diamond represents the screening case, and is classified as most likely a carrier. Access date: 2020/01/09.

CLIR provides single condition tools (SCT) which generate an outcome in four possible categories. The lowest category is “uninformative” and corresponds to cases with an overall score of less than the first percentile. Cases between the 1st and 5th percentiles are “possibly”, the 5th - 20th are “likely”, and scores greater than the 20th percentile are “very likely”. Percentiles are used to standardize scores between conditions, as the varying number of analytes used to produce a score make the raw numbers difficult to compare [3]. In our follow-up program, we assigned these scores to three categories—low, medium, and high risk. The correlation between these two scoring systems are shown in Table 1. When available, dual scatter plots (DSP) are also utilized to recommend follow-up testing, particularly for VLCAD deficiency. When a case on the DSP is in the “indeterminate” quadrant, or the “affected” quadrant, the child is referred for testing, including sequence analysis, while other cases are referred for repeat screens. Representative cases on the DSP are shown in Figure 1. In general, for normal birth weight infants with a positive screen for a condition such as phenylketonuria (PKU) or medium chain acyl-CoA dehydrogenase (MCAD) deficiency, cases which fall into Georgia’s low risk category are resolved with a repeat screen, while all other cases are referred for confirmatory testing. NICU infants and low birth weight (< 2500 g) infants have separate protocols. Calculation of the relevant CLIR scores is done routinely by follow-up staff, and does not delay results being reported to the provider. Reporting abnormal results to the child’s provider includes a review of common symptoms observed in infancy. The presence of concerning symptoms, even for a screen in the low risk category prompts a recommendation for testing, and consultation with an appropriate specialist, as needed. No algorithm can cover all eventualities, nor substitute for professional judgement. As such, clinical specialists serve as consultants to guide follow-up activities in uncertain situations.

While post-analytical tools can be used prospectively to reduce FP results in the laboratory, we have not been able to implement this in our program at this time. Reports are issued to providers automatically by the lab information system, at approximately the same time as they are transmitted to the follow-up database. In the interests of not confusing providers, and creating a system where we are repeatedly telling them to disregard reports that are finalized as abnormal, all results receive follow-up recommendations, regardless of whether subsequent CLIR analysis by the follow-up team reports a high likelihood that the screen will be a FP or likely affected. Our main goal in utilizing CLIR tools for triage of follow-up cases is the appropriate utilization of resources in specialist clinics, as well as easing the burden on families including minimizing confirmatory testing. As a secondary benefit, it has given us familiarity with the tools, and a solid comparison data set for when implementation earlier in the screening process becomes possible.

## 2. Materials and Methods

To evaluate the effectiveness of the current post-analytical tools for the triage of NBS results, we retrospectively extracted from our follow-up database all positive screens for four conditions: Phenylketonuria (PKU), maple syrup urine disease (MSUD), MCAD deficiency, and VLCAD deficiency for a 14-month period (04/01/2018–06/01/2019). These conditions were selected because they are among the most common detected by tandem mass spectrometry in Georgia, and enough screens and true positive (TP) cases were available for comparison. There were no TP cases of MSUD during the study period, however it was included in the population of screen positives as it is a large contributor of FP screens from tandem mass spectrometry (MS/MS) testing. From our database we extracted the data from the initial NBS, the outcome of the case, and whether the outcome was obtained from a repeat NBS or confirmatory testing. We also identified the post-analytical tool results (including DSP results for VLCAD screens) utilized by the follow-up team at the time of the initial triage. As new cases are added to the CLIR database constantly, identifying the CLIR results that were used at the time was important for accurate evaluation. The design of this study was reviewed and approved by the Georgia Department of Public Health Institutional Review Board.

## 3. Results and Discussion

The total number of screens extracted for the study was 538. Initially, all screens were sorted into “isolated” screens, which were those which were abnormal for only a single condition—either PKU (*n* = 51), MCAD deficiency (*n* = 27), or VLCAD deficiency (*n* = 69), and “complex” screens, which were those that were abnormal for at least two conditions detected by MS/MS including at least one of the three targeted conditions (*n* = 391). Infants who expired before an outcome was recorded were excluded and repeat specimens on one child with Citrullinemia Type I post-diagnosis were also excluded. All isolated screens for each condition were included in this study. For the complex screens, they were first split according to birth weight (above or below 2500 g), and then 50 were randomly selected from each group. There was a total of 274 complex screens with birth weights < 2500 g, and 117 complex screens with birth weights > 2500 g. 

**Figure 2:**
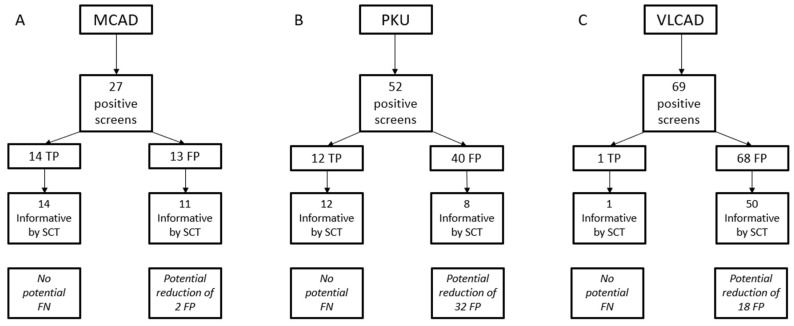
Summary of single condition tool (SCT) results for isolated screens for medium chain acyl-CoA dehydrogenase (MCAD) deficiency (**A**), phenylketonuria (PKU) (**B**), and VLCAD deficiency (**C**). Potential changes in false negative or FP results are what would have been achieved if all informative results were reported out, and all uninformative results were treated as normal.

Summary data for the isolated screens for the three conditions are shown in Table 2, and a flow chart of results for the SCT used for all three conditions is shown in Figure 2. Among MS/MS conditions, MCAD deficiency has one of the highest PPVs, however there is an interference that can cause FP results, particularly in low birth weight infants [12]. Of the 27 positive screens for MCAD deficiency during the study period, 14 were confirmed to be TPs for a PPV of 51.9%. Ten were resolved as normal, and three remain unresolved, but are not immediately concerning for MCAD deficiency. Of the TP cases, all of them had informative scores using the CLIR tool at the time (11 were high risk by Georgia’s categorization and three were medium risk). Of the 13 presumed FP screens, 10 were medium risk, and three were low risk. A repeat NBS was used to resolve two of these cases, while the remaining 25 cases (14 TP and 11 FP) were all referred for confirmatory testing. A single case with a low risk (uninformative) score was referred for confirmatory testing rather than a repeat screen due to the age of the infant (> 2 months), and because the screen came near the end of an extended NICU stay. 

There were 51 total screens which were abnormal only for PKU. By our classification system, there were 32 low risk cases, all of which were recommended to have a repeat screen collected, which were all normal. Three were classified as high risk, which were all confirmed to be TP cases of PKU by confirmatory testing. The remaining 16 cases were classified as medium risk. Three of these were recommended to have repeat screens collected; two due to low birth weight, and one because the mother had a confirmed diagnosis of PKU. These three cases were resolved as normal on the repeat screens. The remaining 13 medium risk cases were resolved with confirmatory testing. Two were normal, two remain unresolved, and nine were abnormal (five hyperphenylalaninemia, and four classic PKU). For isolated screens, the PPV for PKU screening was 23.5%. 

There were 69 isolated screens that were abnormal for VLCAD deficiency. The utilization of post-analytical tools for VLCAD deficiency is a two stage process due to the presence of abnormal biochemical profiles in heterozygous individuals and a robust DSP to discriminate between these conditions. [13] Of the 69 screens reported out as abnormal for VLCAD, one was confirmed to be abnormal, resulting in a PPV for VLCAD deficiency of 1.5% in isolated screens. The single TP case was assigned a medium risk score and proceeded to confirmatory testing after a second screen was abnormal for VLCAD deficiency. At the time of the analysis, the outcome of the DSP for this case was “heterozygous”. Analysis of this same case at the time of manuscript preparation classified the initial screen as VLCAD deficiency by DSP and the repeat (collected approximately 48 h later) as “indeterminate”. This change in classification shows the potential for the database to expand and improve as additional cases are added. Eighteen cases were classified as low risk and resolved as normal by repeat screens. Twenty-three cases had either a medium or high-risk assignment, and by the DSP were either indeterminate or affected. These were all resolved with confirmatory testing, and none were affected. The remaining 27 cases were medium or high risk, but not informative by DSP, and were resolved as normal by a mix of confirmatory testing and repeat NBS. Six positive screens (all medium / high risk and informative by DSP) were confirmed to be carriers. As molecular testing was not pursued in all of these cases, there may be additional carriers in the population classified as unaffected. [14] The goal of NBS is not to identify carriers, thus all cases that were not TPs were classified as FPs, even if the reason for the abnormal biochemical abnormalities was identified. When adding cases to the CLIR database, we only upload those with confirmatory testing, rather than basing it off the CLIR prediction from the NBS. 

The potential improvement in PPV was much better for PKU than for MCAD and VLCAD deficiency. FP screens for the two fatty acid oxidation disorders are often due to carriers with mild biochemical profiles, resulting in analyte deviations that impact only the primary markers and with overlap from TP cases. For PKU, the FP screens are often due to TPN, and even in cases where PKU is the only flagged elevation, other amino acids may have a characteristic pattern that can take greater advantage of the post-analytical tools. More complex profiles provide more chances for the tools to identify distinctive profiles for TP and FP screens. As a specific example, the second most important marker for the identification of PKU is tyrosine (which is decreased). FP screens for PKU do not have decreased tyrosine, while FP screens for MCAD or VLCAD typically have mild elevations of all key analytes, as the same biochemical pathway disturbances are being measured.

While the utilization of post-analytical tools has been effective for the triage of follow-up cases, our goal is to have them implemented during the screening process before the report has been released. It is hoped that by utilizing these tools earlier in the screening process, FP screens can be reduced. This creates less disruption for patients and their families and lowers the workload for participants throughout the NBS follow-up system. For low-risk screens with a high likelihood of being an FP, the ability to resolve them quickly and efficiently is advantageous. Retrospective calculations show significant potential improvements in isolated screens (Table 2). 

The use of post-analytical tools appeared to show more potential improvement in the LBW population compared to the NBW group (Table 3). This may be due to abnormal screens in the smaller babies reflecting normal physiology in this population that cannot be accounted for with static reference ranges. CLIR’s adjustments for age at collection and BW can more smoothly account for this variation and guide follow-up in this population. Due to TPN interference, particularly in premature infants, the complex screens contain a disproportionately large number of abnormalities for amino acid disorders. Based on Georgia’s algorithms in place, 30 of the NBW screens and 29 of the LBW screens were resolved with repeat screens. In the NBW population, of the 42 screens that provided an informative score, 24 of these were abnormal only for PKU. In the LBW population, nine of the 18 screens were abnormal only for PKU. An inspection of the underlying results showed that most of these cases triggered outliers for tools designed to detect MSUD and other amino acid disorders. The creation of a tool or algorithm to flag results that are likely due to TPN could provide further assistance in the triage of these cases. Improvements in PPV from the complex screen population could not be calculated, as no TP cases for the targeted conditions originated from this group. Eliminating all screens with uninformative results could have resulted in 16% fewer abnormal screens in NBW infants and 64% fewer screens in the LBW cohort. 

Our end goal is to implement CLIR as part of the testing algorithm to reduce FP screens at the laboratory level. The retrospective data we have obtained on the performance of CLIR tools with our screens in Georgia will be helpful as we move forward with this. While algorithms will still need to be developed and validated prior to complete implementation, studies such as this one link outcome to post-analytical tool results at a local level, which provides important information for decision making. Simple steps, such as making the decision to not report all uninformative cases could improve the PPV dramatically, particularly for amino acid disorders where TPN is a common confounding factor. In this study, not reporting uninformative cases for PKU would have improved the PPV of isolated screens from 23.5% to 63.2%. This means 32 cases would not have been reported, and the child would not have required any further medical intervention based on NBS results. In a situation where most infants do not have a disorder identifiable by NBS, resolving cases as normal as early in the process as possible is important for an efficient system.

## 4. Conclusions

Post-analytical tools have been beneficial for the NBS follow-up program in Georgia. When utilized for follow-up purposes in the three disorders selected for this study, all TP cases had an informative score with the appropriate post-analytical tool. This allowed for prompt follow-up testing, and correct usage of repeat NBS to resolve cases with a lower likelihood of being a TP. Importantly, this has provided evidence that CLIR’s tools appropriately identified cases that required follow-up attention. CLIR does not underestimate results, making it unlikely that a TP will be missed if it would have been identified using appropriately set cutoffs. Examination of these screens retrospectively has demonstrated that screens with multiple mild abnormalities introduce a disproportionate amount of FP results into the NBS system, and post-analytical tools can be an effective filtering method. For the three conditions included in this study, no confirmed abnormal results were on complex screens (defined as those abnormal for less than one condition detected by MS/MS). In many cases, there will be logical patterns to complex screens in true abnormals (multiple long chain acylcarnitine elevations can be present), and profiles typical of “complex FPs” can also be identified. Post-analytical tools can account for these FP screens, however additional granularity may be helpful, as not all FP results are the same. In particular, the common FP profiles for LBW infants differ from NBW infants, as seen by the differences in performance with complex screens in this study, and in the results obtained using post-analytical tools in each cohort. 

The utilization of post-analytical tools in Georgia has allowed for effective triage of results, reduced the amount of confirmatory testing needed, standardized the communication of NBS results with providers and given a path forward for the implementation of these tools earlier in the screening process. These tools have provided structure for the follow-up of large groups of conditions, with expanding teams of specialists. The standardization of risk scores has been helpful for clinicians, rather than needing to keep track of multiple cutoff values, and scales of abnormal results, this information can be communicated quickly and effectively utilizing the CLIR score, and categorization. This model could be utilized in many state NBS labs, as there are common screening kits utilized, allowing for shared development work and tool maintenance if desired. The proof of concept shown here identifies another possible area of implementation for post-analytical tools if the IT requirements to utilize CLIR tools before results are reported cannot be easily implemented locally.

## Figures and Tables

**Table 1:** Correlation between Collaborative Laboratory Integrated Reports (CLIR) single condition tool guidelines and Georgia newborn screening (NBS) follow-up triage risk levels.

CLIR Single Condition Tool Guideline	Georgia Follow-up Triage Level
Uninformative	Low Risk
Possibly	Medium Risk
Likely	
Very Likely	High Risk

**Table 2:** Summary results for isolated screens for MCAD deficiency, VLCAD deficiency, and PKU. *Potential positive predictive values (PPV) considers performance if all uninformative cases had not been reported.

Condition	Abnormal Screens	TP Cases	TP Cases Informative	CLIR Identification Rate	CLIR Uninformative	Actual PPV	Potential PPV
**MCAD**	27	14	14	100%	3	51.90%	58.3% * (14/24)
**PKU**	51	12	12	100%	32	23.50%	63.2% * (12/19)
**VLCAD**	69	1	1	100%	18	1.50%	2.0% * (1/51)

**Table 3:** Correlation and performance of CLIR tools with complex screens. NA = Not applicable; there were no TP cases identified in either cohort.

	Number of Screens Sampled	Average # of Abnormal Conditions Per Screen	TP Cases	CLIR Identified TP	CLIR Uninformative
BW < 2500 g	50	3.26	0	NA	32
BW > 2500 g	50	3.04	0	NA	8
